# Effect and benefit of percutaneous coronary intervention in chronic total occlusion on ventricular repolarization: QT correction and dispersion

**DOI:** 10.25122/jml-2022-0207

**Published:** 2022-08

**Authors:** Ameen Abdulhasan Al Alwany

**Affiliations:** 1 College of Medicine, University of Baghdad, Baghdad, Iraq

**Keywords:** QT interval, QTc dispersion, chronic total occlusion

## Abstract

ST segment, T wave changes, QT interval changes, and QTc dispersion are among the parameters used to diagnose ischemic heart disease. The increase in the QT dispersion can be caused by myocardial ischemia, among other heart diseases, whereas cardiac diseases such as coronary artery disease (CAD) can be diagnosed by observing an abnormally high QTc dispersion. This study aimed to evaluate the variations in the QTc dispersion (depolarization and repolarization) of surface electrocardiography as a result of percutaneous coronary intervention (PCI) in patients with chronic total occlusion. This study took place in the Iraqi Center for Heart Disease from October 2020 to February 2021. 110 patients who suffered from chronic occlusion of the coronary artery and underwent PCI revascularization were examined. Twelve-lead electrocardiograms were recorded at the time of admission (12 hours before intervention) and more than one hour after the intervention. The measured ECG parameters included corrected QT interval durations and corrected QT dispersion in both pre and post-PCI electrocardiograms, and their values were compared. The average corrected QT interval and QTC dispersion changed significantly before and after the percutaneous coronary intervention. Performing percutaneous coronary intervention on patients who suffer from coronary artery total occlusion shows a major reduction in the corrected QT dispersion.

## INTRODUCTION

A cycle of ventricular electrical activity, including depolarization and repolarization, is often represented by the QT interval. The QT interval is measured considering the time between the start of the QRS wave and the end of the T wave. Many diseases, such as long QT and short QT syndromes, can be diagnosed by observing the changes in the length of this specific interval [1]. A long QT interval is referred to as repolarization abnormality. This repolarization abnormality can be caused by the changes in the ion distribution due to abnormal opening and closing durations of the relative ion channels, which can result from congenital defects of said channels [2]. When comparing measurement technologies, it is much easier to replicate the results of measuring QT intervals using faster paper speed recordings. However, using a slower paper speed leads to a longer QT interval, and the T wave amplitude also increases. Therefore, it becomes hard to find the exact balanced value to use, even though most researchers often use the typical speed for a clinical 12-lead ECG which is a paper speed of 25 mm/s [3, 4].

Heart rate correction: Bazett's formula: QTc=QT/√(RR).

Bazett's formula is one of the most famous and frequently used formulas to measure QT interval correction. Despite being discovered and practiced for around 80 years, it has gained some criticism from practitioners as to whether it is a valid tool to measure heart corrections. Some claim that this formula is generally only appropriate when the heart rate is 100 beats/min, whereas the formula cannot be considered to entirely compensate for the heart rate if it is still associated with the heart rate after the correction procedure [4, 5]. This mis-compensation results from the overcorrecting at higher heart rates. To solve this problem, another linear correction formula has been developed to be used at higher heart rates. In addition, researchers found that heart rate correction at the level of individual QT intervals and measuring the changes in QTc max can be more important than adjusting the QT dispersion. When comparing five commonly used methods, the results were similar to that of Bazett's formula, and this data was acquired from several reports, including the Rotterdam review [4–6].

However, a QT interval correction formula has been recently introduced based on the changes in heart rate that are exercise-induced, and its results look promising [5, 7]. When introducing new drugs and a shorter regimen, the Fredericia formula is the ideal formula to be used, among three other formulas: Bazett, Framingham, and Hodges.

The QTcF, like Bazett QTc, can be managed and calculated manually or automatically on the ECG machine.

Because the T wave morphology is not fixed between leads, it is important to analyze multiple leads and choose the longest QT interval to obtain reliable results. One suitable method to evaluate the QT interval prolongation is by inspecting the relationship between the QT interval and the distance between 2 R waves [2–7]. A QT interval is considered prolonged if it ends after the halfway point of the R-R waves. Otherwise, it is said to be non-prolonged [5].

As for the corrected QT interval, “QTc” is a term used to assess the prolongation with respect to the heart rate. Bradycardia or a slow heart rate results in a prolonged QT interval. QTc is calculated by dividing the measured QT interval by the square root of the R-R interval. It is expected for the normal values of both the QT interval and QTc interval to vary upon age and between genders. However, corrected QT must have a value less than 440 ms [3–9].

Certain angiographic and clinical standards determine the success or failure of revascularization. If the final minimum stenosis diameter is reduced to less than 10 percent, the angiographic standard is met and considered successful [10]. For the procedure to be completely successful, the patient must have angiographic success and no further complications such as myocardial infarction (MI), emergency coronary artery bypass graft (CABG), or death. As for clinical success, it is measured by the insurance of procedure success, as mentioned above, in addition to the relief of symptoms of previously existing myocardial ischemia [6–11].

## Material and Methods

The following cross-sectional study was conducted in the Iraqi Center of Heart Disease in cooperation with the Baghdad College of Medicine from October 2020 to February 2021. This study included 110 consecutive patients suffering from chronic total occlusion enrolled based on specific criteria.

Inclusion criteria: angiographic evidence of significant coronary artery lesions, chronic total occlusion, and effective angiographic PCI.

Exclusion criteria: patients with acute coronary syndrome, ventricular pacing, or electrolyte disorders. Also, patients with QRS duration longer than 0.12 seconds or whose QT interval was not available to measure for 8 leads. In addition, patients who were not in sinus rhythm, who took anti-thrombosis medicine, and those who needed CABG or repeated PCI within 24 hours of the procedure were also excluded.

All patients were examined using a history, clinical evaluation, and the initial coronary angiogram (CAG). It is only necessary to perform PCI if the vessel is severely stenosed. For example, the left main coronary artery (LMCA) received a “50%” rating, while the left anterior descending artery (LAD), left circumflex artery (LCX), and right coronary artery (RCA) received a “70%” rating. Vessels with previously implanted patent stents were considered common, even if there were multiple patent stents. Some cases involved stent restenosis (ISR), characterized as stent restenosis of 50% or more of previously implanted stents rather than a new de novo coronary lesion. Echocardiography was done for all patients, and a normal ejection fraction was defined as more than 55%. According to this definition, most of the study population had a normal ejection fraction.

### Coronary angiography

The coronary angiography and angioplasties took place at the catheterization lab of the Iraqi Center for Heart Diseases. PCI was done through the right femoral approach and executed according to standard techniques in most patients. In addition, blood urea, serum creatinine, serum potassium, calcium and magnesium, blood sugar, and viral screen for hepatitis B and C and HIV were done before the intervention. Furthermore, all patients had an echocardiographic assessment before PCI, and most had normal ejection fraction (defined as more than 55%).

### The ECG

Standard 12-lead ECGs were documented at the time of admission (nearly 12 hours) before PCI and more than one hour after PCI. The electrocardiograph took into account three or six standard leads simultaneously using a paper speed of 25 mm/s and a gain of 10 mm/mV. To measure the QT interval, the time from the start of the Q wave until the end of the T wave was considered in each of the 12-ECG leads. The average of and sometimes the highest (if T wave in other cycles was not measurable) of the QT interval in three consecutive cycles in each lead was taken, and a minimum of 8 leads were taken from those patients with ECG of poorly identifiable T wave end.

After magnification, the QT interval results were analyzed. Two measurements were taken, one before and one after PCI. A ruler was used to calculate the QT interval from the start of the QRS complex to the end of the T wave. The point of return to the isoelectric line was established as the end of the T wave. The end of the T wave was noted by the nadir between the two waves when a T wave is interrupted by a U wave. However, some recordings of QT measurements were omitted from the study in case of very low voltage (0.1 mV).

In addition, the Bazett formula was implemented to correct the exclusion of the effect of heart rate on the QT intervals. The Bazett formula: QTc=QT/square root of RR interval. The difference between the maximum and the minimum amplitude of the corrected QT interval is considered to be the QT dispersion.

### Methods employed in measuring QT intervals

- Lead II or V5-6 is used to obtain the QT interval;
- Measuring should take into consideration numerous consecutive beats and record the maximum interval;
- The measurements should take account of the U waves intercepting with T and larger than 1mm;
- The measurements do not include the U waves less than 1 mm or not intercepting with T wave;
- To identify the end of the T wave, the maximum slope intercept method is implemented [5].

### Statistical analysis

SPSS-25 (Statistical Packages for Social Sciences- version 25) was used to analyze the obtained data. Values such as frequency, mean, standard deviation, percentage, and range were utilized to represent the data. The significance was measured by different approaches such as the student's t-test (for the difference between two independent means), paired- t-test (for the difference between two dependent means), or ANOVA test (for difference among more than two independent means). The difference was considered statistically significant if the P value was equal to or less than 0.05 [6].

## Results

93 out of 110 patients were males (84.5%), whereas only 17 were females (15.5%). The age of these patients was in the range of 36 years to 79 years. 36% were 60–69 years old, 35.5% were 50–59, and 16.4% were 40–49. Another group of patients was >70 years old, constituting 8.2% of the study population, and 3.6% of patients were <40 years old (Table 1).

**Table 1:** Distribution of patients according to age and gender.

		No	%
**Age (years)**	<40 years	4	3.6
	40–49	18	16.4
	50–59	39	35.5
	60–69	40	36.4
	≥70 years	9	8.2
	Mean±SD (Range)	57.0±9.1 (35–78)	
**Gender**	Male	93	84.5
	Female	17	15.5

Patients in the left anterior descending artery (LAD) community made up 74.5% of the total, and there were major variations in QTc interval and QT dispersion before and after PCI. The left circumflex artery (LCX) community included 50 patients (45.5%), and there were major variations in QTc dispersion before and after PCI. The right coronary artery (RCA) group consisted of 46 patients (41%), with major variations in QTc dispersion before and after PCI. There were 55 patients (50%) with single vessel disease, 32 (35.5%) with two-vessel disease, and 16 (14.5%) with three-vessel disease (Table 2).

**Table 2:** Distribution of patients according to the number of diseased coronary vessels.

		No	%
**Number of stenosed vessels**	Single VD	55	50.0
	Two VD	39	35.5
	Three VD	16	14.5
**LAD**	LAD stenosis	82	74.5
	Not	28	25.5
**RCA**	RCA stenosis	46	41.8
	Not	64	58.2
**LCX**	LCX stenosis	50	45.5

The mean value of QTc dispersion before PCI was 49.3±21.3 (7–107), and post PCI, 24.8±12.7 (2–64) (Table 3). The results show that the difference in QTc dispersion reduction is statistically significant after PCI.

**Table 3:** QT dispersion pre and post-PCI.

		Pre-PCI		Post-PCI	
		No	%	No	%
**QTD**	<10 milliseconds	1	0.9	13	11.8
	10–19	5	4.5	26	23.6
	20–29	9	8.2	36	32.7
	30–39	19	17.3	21	19.1
	40–49	29	26.4	9	8.2
	50–59	18	16.4	4	3.6
	60–69	13	11.8	1	.9
	70–79	3	2.7	-	-
	80–89	9	8.2	-	-
	90–99	-	-	-	-
	≥100 milliseconds	4	3.6	-	-
	Mean±SD (Range)	49.3±21.3 (7–107)		24.8±12.7 (2–64)	

P=0.0001 (Significant difference between the pre-and post-PCI mean value of QTD using paired t-test at 0.05 level).

**Figure 1:**
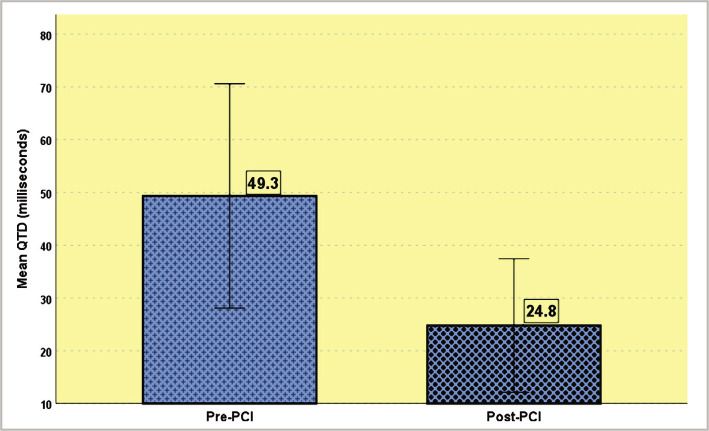
Mean QTD pre and post-PCI.

Before PCI, the maximum QTc interval was 425.4±38.8 (360–540), and the minimum QTc interval was 376±24.2 (310–436). After PCI, the maximum QTc interval was 414.2±28.8 (336–488), and the minimum QTc interval was 389.5±27.0 (279–448) (Table 4, Figure 1)

**Table 4:** The longest and shortest QTc interval before and after PCI.

	Mean±SD (range)
**Max Pre-PCI QT1**	425.4±28.8 (360–540)
**Min Pre-PCI QT2**	376.1±24.2 (310–436)
**Max Post-PCI QT1**	414.2±28.8 (336–488)
**Min Post-PCI QT2**	389.5±27.0 (297–448)

There was no significant difference in the degree of QTc dispersion reduction between males and females (Table 5).

**Table 5:** Correlation between age and sex with the mean QTc dispersion pre- and post-PCI.

		No	Pre-QTD (milliseconds)	Post-QTD (milliseconds)	P-value
			Mean±SD	Mean±SD	
**Age (years)**	<40 years	4	42.8±8.6	13.5±5.7	0.024*
	40–49	18	47.1±12.6	27.4±12.9	0.0001*
	50–59	39	52.8±24.3	27.2±12.9	0.0001*
	60–69	40	49.0±22.2	23.8±12.5	0.0001*
	≥70 years	9	43.6±21.4	18.1±10.1	0.002*
	P-value		0.692	0.086	
**Gender**	Male	93	50.1±20.1	25.6±12.4	0.0001*
	Female	17	45.1±27.2	20.2±13.7	0.0001*
	P-value		0.376	0.104	

* – significant for p less than 0.05.

QTc dispersion was significantly correlated with the amount of coronary vessels that had atherosclerosis. We observed a significant positive correlation between QTc dispersion variation and the number of vessels involved. Accordingly, the change in QTc dispersion was found in patients with SVD as a pre-PCI value of 49.1±21 (Table 6) and a post-PCI value of 22.6±12.7. The reduction in QTc dispersion post PCI for double VD was also significant. Likewise, those with 3 vessels disease had a significant reduction in QTc dispersion post PCI.

**Table 6:** QTc dispersion changing status with respect to the number of coronary arteries involved.

		No	Pre-QTD (milliseconds)	Post-QTD (milliseconds)	P-value
			Mean±SD	Mean±SD	
**Number of stenosed vessels**	Single VD	55	49.1±21.0	22.6±12.7	0.0001*
	Two VD	39	48.4±20.6	26.3±11.1	0.0001*
	Three VD	16	52.6±24.6	28.4±15.4	0.0001*
	P-value		0.792	0.180	

* – significant difference between two dependent means using paired-t-test at 0.05 level.

## Discussion

When the QT dispersion increases, it indicates variant ventricular repolarizations that differ, warning about severe ventricular arrhythmias [11]. This risk of prolonged QT dispersion becomes more serious in patients with hypertrophic cardiomyopathy, myocardial infarction, and malignant ventricular arrhythmias [12]. Consequently, effective management of acute myocardial infarction and ventricular arrhythmias would lead to a decrease in the QT dispersion, which can occur via successful reperfusion after the lysis of a thrombus, or revascularization via angioplasty and CABG grafting, and more effectively if it were associated with aneurysmectomy. Several clinical practices studied how QT dispersion can be affected by myocardial ischemia [12–14].

Concerning the ischemia, the transient type leads to prolongation of the QT dispersion, whereas acute ischemia (caused by balloon inflation) increases the QT dispersion in a more prominent manner. However, in that case, the prolongation can be reversed upon reperfusion [13].

Michelucci *et al*. [7] established that the ventricular recovery pattern is less consistent in the cases of ischemia and reperfusion [15]. A major reduction in QT dispersion can be useful as an electrocardiographic indication for efficient blood flow restoration. In one analysis, the QT dispersion after primary PCI was much less than the QT dispersion after thrombolysis. This difference can be accredited to more recovered myocardium in the primary PCI group since the QT dispersion essentially refers to the volume of viable myocardium in the infarct area. For this reason, QT dispersion is a “viability marker” in patients suffering from chronic Q-wave myocardial infarction. As for chronic stable angina patients, they could show irregular QT dispersion due to ongoing ischemia, prior myocardial injury, or both. Several studies discuss the variation in QT dispersion in patients with chronic ischemia due to revascularization, but it remains debatable since the findings are not always consistent. In their study, Choi *et al*. [8] showed that the patients with no history of myocardial infarction revealed a decrease in the QT dispersion even after 30 days of undergoing PCI [2].

Similarly, Aydinlar *et al*. [9] published a study illustrating that percutaneous trans-luminal coronary angioplasty results in instantaneous QT dispersion.

Our study showed a reduction of QTc dispersion as early as one hour after successful PCI. The collected data clarify that ventricular repolarization and QTc dispersion are changed due to chronic ischemia in patients with stable angina [16, 17]. Therefore, successful myocardial revascularization in cases of chronic stable angina results in a substantial decrease in QTc dispersion as early as one hour after the procedure.

These results agree with the proposition that a careful study of QTc dispersion from the surface ECG will identify inconsistency of ventricular refractoriness due to ischemia. The high QT dispersion values in our patients indicate that they were a high-risk population, and almost all had significant ischemia. Our research also found that all patients, including those with normal QTc dispersion, had a substantial reduction in QTc dispersion (those with 50 or less than 50 ms dispersion). As a consequence, revascularization decreased QTc dispersion and alleviated ischemia. In addition, no significant difference was recorded in the baseline characteristics of these classes, and the reason remains ambiguous. As a result, future studies should include a greater sample size in each category to explore this problem more thoroughly [15–17].

The average age of the patients in our sample was 57.091 years. The age group 41–60 years were the most frequent. Yilmaz *et al*. [10], as well as Tikiza *et al*. [11], found almost identical mean ages, which are comparable to the findings of the current study. In our study, there were slightly more male patients than in other studies (84% *versus* 76% in other countries, likely due to social factors).

### Variation of the QTc dispersion after percutaneous coronary intervention

In this research, the mean QTc dispersion was 49.3 msec before PCI and 24.8 msec after PCI, which was statistically important. The reduction in mean QTcd after PCI was 24.5 msec, almost identical to what Alasti *et al*. observed [12]. In their analysis, QTc dispersion was 80.40 ms before PCI and 60.40 ms after PCI. Others also recorded a reduction in QTc dispersion following PCI [8, 13–15]. This decrease in QTc dispersion suggests good revascularization following PCI and a reduction in ischemia pressure due to reperfusion. In our research, we discovered that among 110 patients, 55 had one vessel disease (50%), 39 had double vessel disease (35.5%), and 16 had triple vessel disease (14.5%). The increased number of patients with three-vessel disease who underwent PCI in our sample (in contrast to other studies) reflects our patients' aversion to CABG surgery and preference for PCI. Prolonged QT dispersion is directly associated with an increase in the number of affected vessels. As a result, the shift in QTc dispersion for single vessel disease (SVD) was 26.6±8.3 ms, while for double vessel disease were (DVD) 22.1±9,5 ms, and for three-vessel disease (TVD) were 24.2±9.2ms (p-value=0.180). The percent reduction in QTcd was 52,6±21,2 percent in cases of single vessel disease, 43.3±21,5 percent in cases of two-vessel disease, and 46.5±18.4 percent in cases of three-vessel disease. As the number of vessels involved increases, so does the decrease in QTc dispersion after PCI, but the difference is negligible (p-value=0.103). A difference in the QTc dispersion among patients based on the different coronary arteries was recorded [15–17].

As previously stated, we discovered a correlation: the QTc dispersion variation was directly related to the quantity of vessels involved in our research. In addition, an obvious positive link between the shift in QTc dispersion and the growing number of vessels involved was observed, and this importance was decided according to Spearman's correlation test. The most significant discovery of this study is that effective percutaneous coronary intervention reduced prolonged QTc dispersion (PCI). This was also discovered in the studies of Mohammed Alasti *et al*. [12], Shafiqulislam *et al*. [16], and all of the studies listed in the discussion [16–19].

These results represent a key representation of successful reperfusion and are also essential for the prognosis of post PCI patients.

## Conclusion

PCI greatly decreases QTc dispersion in patients with chronic complete occlusion. This is determined by the magnitude of the coronary artery stenosis and the number of diseased vessels. Reduced QTc dispersion is a positive indication of active PCI since it means successful reperfusion, which has a good prognostic benefit.
